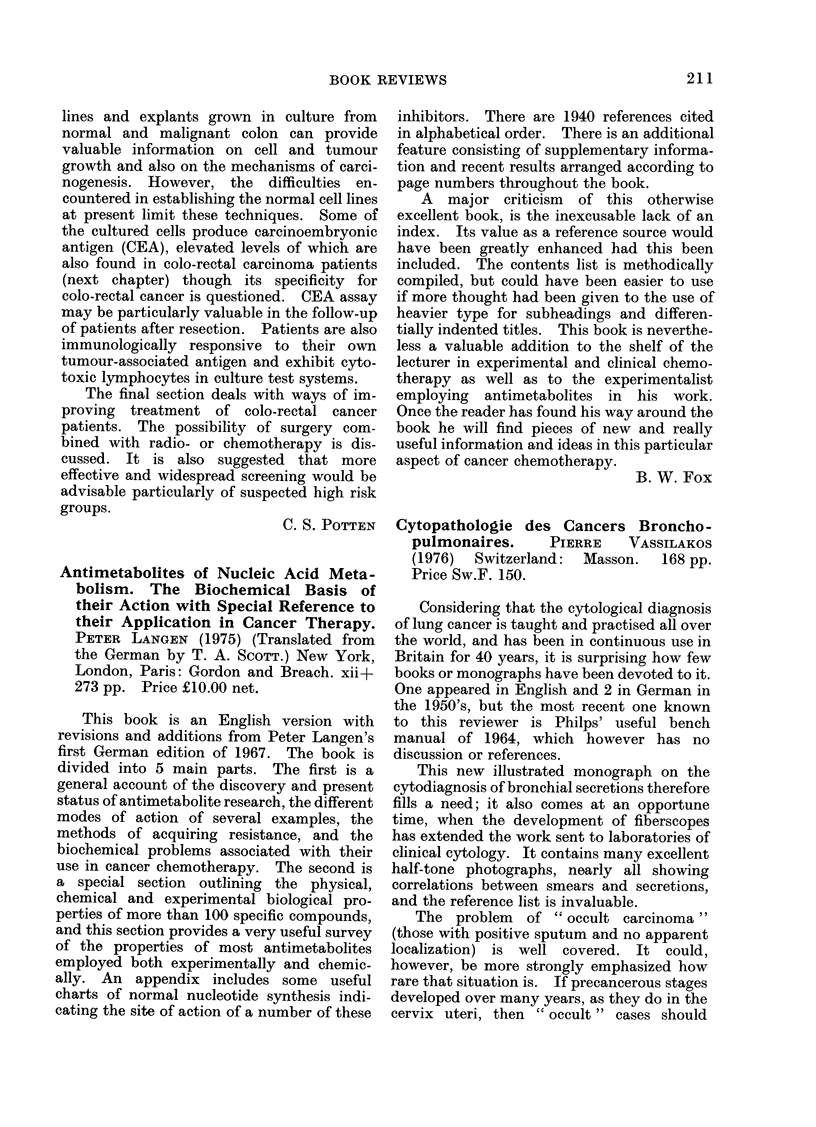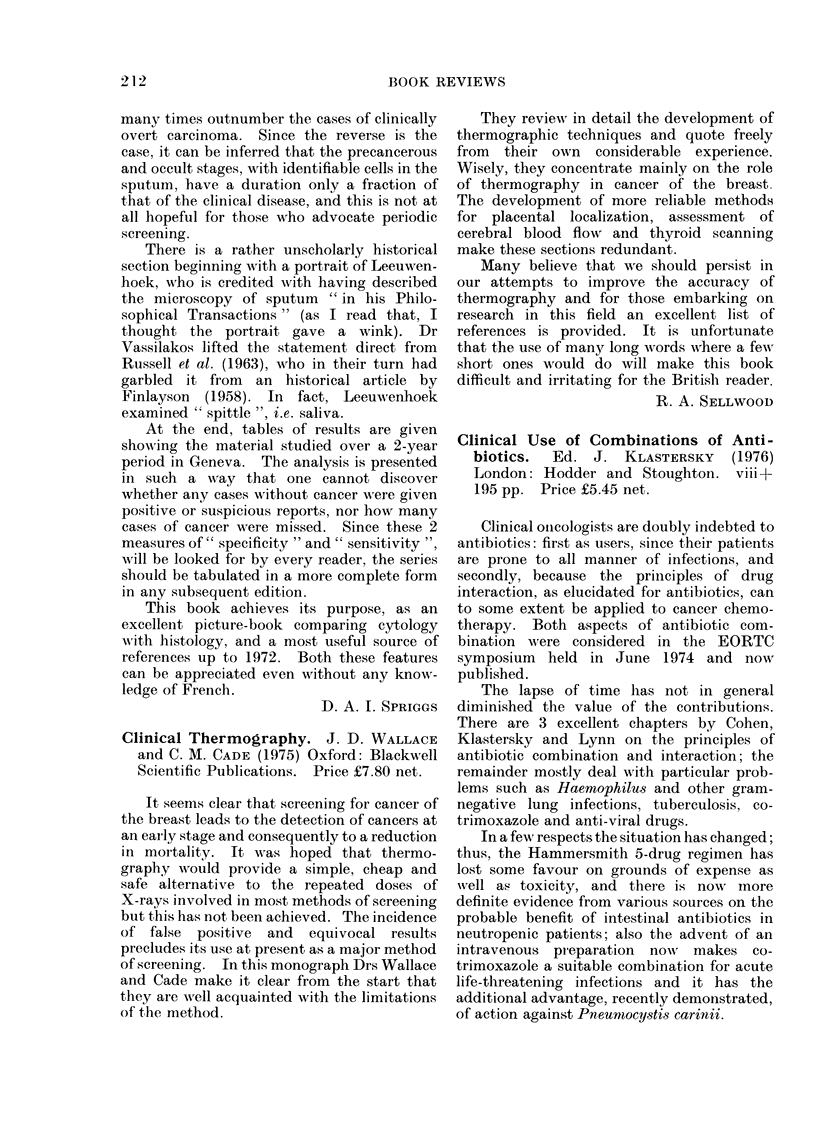# Cytopathologie des Cancers Bronchopulmonaires

**Published:** 1976-08

**Authors:** D. A. I. Spriggs


**Cytopathologie des Cancers Bronchopulmonaires.** Pierre Vassilakos (1976) Switzerland: Masson. 168 pp. Price Sw.F. 150.

Considering that the cytological diagnosis of lung cancer is taught and practised all over the world, and has been in continuous use in Britain for 40 years, it is surprising how few books or monographs have been devoted to it. One appeared in English and 2 in German in the 1950's, but the most recent one known to this reviewer is Philps' useful bench manual of 1964, which however has no discussion or references.

This new illustrated monograph on the cytodiagnosis of bronchial secretions therefore fills a need; it also comes at an opportune time, when the development of fiberscopes has extended the work sent to laboratories of clinical cytology. It contains many excellent half-tone photographs, nearly all showing correlations between smears and secretions, and the reference list is invaluable.

The problem of " occult carcinoma " (those with positive sputum and no apparent localization) is well covered. It could, however, be more strongly emphasized how rare that situation is. If precancerous stages developed over many years, as they do in the cervix uteri, then " occult " cases should many times outnumber the cases of clinically overt carcinoma. Since the reverse is the case, it can be inferred that the precancerous and occult stages, with identifiable cells in the sputum, have a duration only a fraction of that of the clinical disease, and this is not at all hopeful for those who advocate periodic screening.

There is a rather unscholarly historical section beginning with a portrait of Leeuwenhoek, who is credited with having described the microscopy of sputum " in his Philosophical Transactions " (as I read that, I thought the portrait gave a wink). Dr Vassilakos lifted the statement direct from Russell *et al.* (1963), who in their turn had garbled it from an historical article by Finlayson (1958). In fact, Leeuwenhoek examined " spittle ", *i.e.* saliva.

At the end, tables of results are given showing the material studied over a 2-year period in Geneva. The analysis is presented in such a way that one cannot discover whether any cases without cancer were given positive or suspicious reports, nor how many cases of cancer were missed. Since these 2 measures of " specificity " and " sensitivity ", will be looked for by every reader, the series should be tabulated in a more complete form in any subsequent edition.

This book achieves its purpose, as an excellent picture-book comparing cytology with histology, and a most useful source of references up to 1972. Both these features can be appreciated even without any knowledge of French.

D. A. I. SPRIGGS